# Aluminum exclusion and aluminum tolerance in woody plants

**DOI:** 10.3389/fpls.2013.00172

**Published:** 2013-06-12

**Authors:** Ivano Brunner, Christoph Sperisen

**Affiliations:** Forest Soils and Biogeochemistry, Swiss Federal Institute for Forest, Snow and Landscape ResearchBirmensdorf, Switzerland

**Keywords:** acid soils, adaptation, aluminum, organic acids, tolerance, resistance, toxicity

## Abstract

The aluminum (Al) cation Al^3^^+^ is highly rhizotoxic and is a major stress factor to plants on acid soils, which cover large areas of tropical and boreal regions. Many woody plant species are native to acid soils and are well adapted to high Al^3^^+^ conditions. In tropical regions, both woody Al accumulator and non-Al accumulator plants occur, whereas in boreal regions woody plants are non-Al accumulators. The mechanisms of these adaptations can be divided into those that facilitate the exclusion of Al^3^^+^ from root cells (exclusion mechanisms) and those that enable plants to tolerate Al^3^^+^ once it has entered the root and shoot symplast (internal tolerance mechanisms). The biochemical and molecular basis of these mechanisms have been intensively studied in several crop plants and the model plant *Arabidopsis*. In this review, we examine the current understanding of Al^3^^+^ exclusion and tolerance mechanisms from woody plants. In addition, we discuss the ecology of woody non-Al accumulator and Al accumulator plants, and present examples of Al^3^^+^ adaptations in woody plant populations. This paper complements previous reviews focusing on crop plants and provides insights into evolutionary processes operating in plant communities that are widespread on acid soils.

## INTRODUCTION

Aluminum (Al) is a prevalent constituent of most soils and is one of the major stresses to plants in acid soils. Most of the Al in soils is incorporated into aluminosilicates and other precipitated forms, which are harmless to plants. Under acid soil conditions, these minerals solubilize to a limited extent, and the toxic ion Al^3^^+^ is released into the soil solution (Kinraide, 1997). This form of Al is capable of inhibiting root growth and damaging cells at the root apex, which is the most sensitive part of the root to Al^3^^+^ (Ryan et al., 1993; Kochian, 1995). However, the mechanism underlying Al^3^^+^ toxicity is not clearly understood. Because Al^3^^+^ can interact with a number of extracellular and intracellular structures, many different mechanisms of Al^3^^+^ toxicity have been proposed. These mechanisms include modification of the cell wall, disruption of the plasma membrane and transport processes, interruption of signaling pathways, and Al^3^^+^ binding to the DNA (Kochian et al., 2005).

**FIGURE 1:**
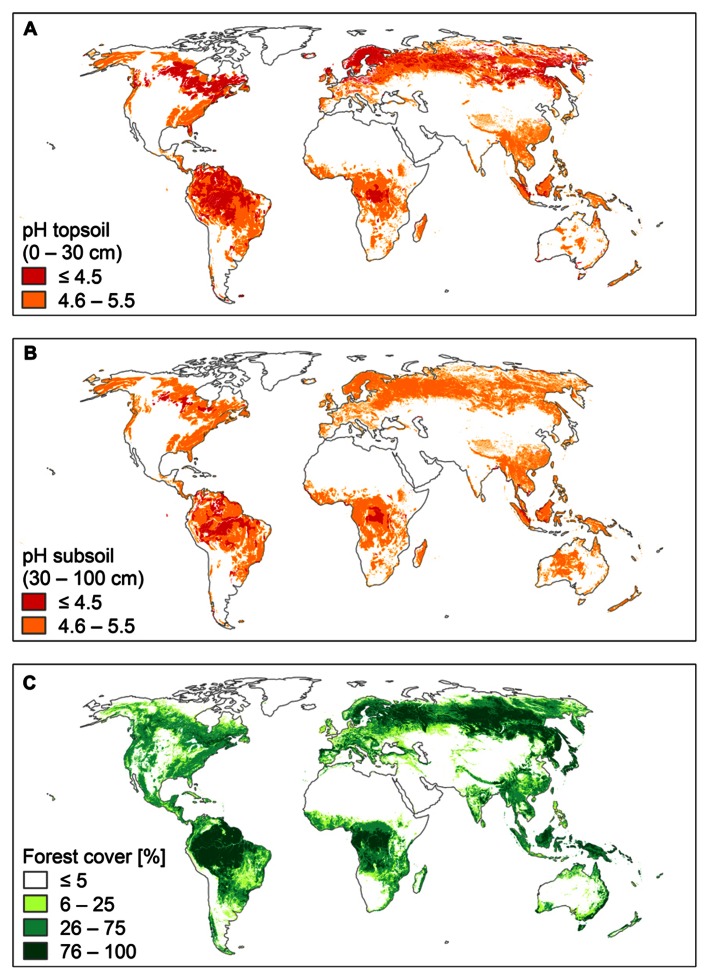
**World acid soils and world forests.** **(A)** pH of topsoil (0–30 cm), **(B)** pH of subsoil (30–100 cm), and **(C)** forest cover. Soil pH is presented in two classes: pH ≤ 4.5 (strongly acid soils) and pH 4.6–5.5 (moderately acid soils). Data were retrieved from the Harmonized World Soil Data Base (FAO/IIASA/ISRIC/ISS-CAS/JRC, 2012). Forest cover is presented in four classes: ≤5, 6–25, 26–75, and 76–100%. Data were retrieved from the Food Insecurity, Poverty and Environment Global GIS Database (FGGD; FAO and IIASA, 2007).

Al^**3**^^+^ toxicity is an important research topic, because many crop plants are susceptible in acid soils, and their growth and yield are limited by high Al^3^^+^ conditions. Less attention is paid to native plant communities, which tolerate acid soil conditions over large areas in different biomes. Acid soils occupy about 30% of the ice-free land area in the world and primarily occur in the humid tropics and the boreal region. Large parts of these soils (about 67%) are covered by forests and woodland (von Uexküll and Mutert, 1995; **Figure 1**). The biodiversity and high biomass production of both tropical and boreal forests suggest that their plants are not affected by Al^3^^+^ toxicity.

Al^3^^+^ toxicity may occur in forests that are exposed to acid deposition derived from air pollutants (Cronan and Schofield, 1979; Ulrich et al., 1980; Larssen et al., 2006). On sensitive sites, acid deposition accelerates soil acidification and leads to increased Al^3^^+^ concentrations in the soil solution (Blaser et al., 1999; Fowler et al., 1999). Recently, it was found that soils, affected by acid deposition, showed signs of recovery due to the reduction in sulfate deposition (Stoddard et al., 1999; Evans et al., 2001). However, inputs of nitric acid and ammonia continue to alter the chemistry of forest soils and are likely to promote acidification (Graf Pannatier et al., 2011; Zang et al., 2011). Acid soils characteristically contain high amounts of Al^3^^+^ and low amounts of the base cations (BC) Ca^2^^+^, Mg^2^^+^, and K^+^, which are important plant nutrients. Since Al^3^^+^ and BC interact at the plasma membrane surface, it is not the soil Al^3^^+^ concentration alone that determines the plant responses to Al^3^^+^ exposure (Sverdrup and Warfvinge, 1993; Cronan and Grigal, 1995; Kinraide, 2003). A ratio of Ca^2^^+^/Al^3^^+^ or BC/Al^3^^+^ in the soil solution lower than 1 is widely used as an ecological indicator for potentially adverse effects of Al^3^^+^ stress and nutrient imbalance on tree growth. Alternative indicators are based on the Al and Ca concentrations in fine roots and provide information on the availability of toxic Al^3^^+^ in the soils (e.g., Brunner et al., 2004; Richter et al., 2007; Vanguelova et al., 2007).

## Al^3+^ EXCLUSION AND Al^3+^ TOLERANCE MECHANISMS

The mechanisms conferring resistance to Al^3^^+^ have been the focus of intensive research in crop plants and in the model plant *Arabidopsis*. Many different mechanisms have been suggested, but for most of them, the supporting genetic and physiological evidence is not provided. Therefore, these mechanisms have to remain speculation or hypotheses until supporting data is provided. One exception is the Al^3^^+^-induced efflux of organic acids from roots, which has been demonstrated to be a major Al^3^^+^ resistance mechanism in several plant species (Delhaize et al., 1993, 2007).

**FIGURE 2:**
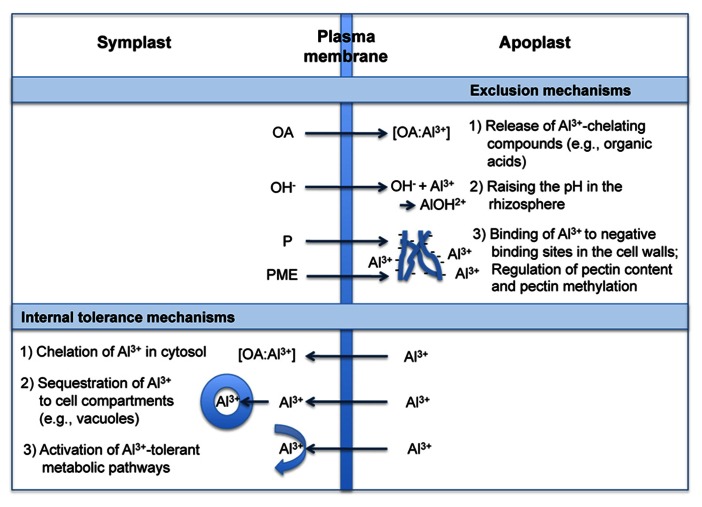
**Mechanisms of plant roots to deal with Al^**3**+^ (modified from Jones and Ryan, 2003; Inostroza-Blancheteau et al., 2012).** OA, organic acids; P, pectin; PME, pectin methylesterase.

Following Ryan and Delhaize (2010) and Horst et al. (2010), the term “Al^3^^+^ resistance” is used here as a plant property that allows a plant to grow with little or no injury under elevated Al^3^^+^ conditions. The potential mechanisms conferring resistance to Al^3^^+^ can be broadly divided into those that exclude Al^3^^+^ from the root symplast (exclusion mechanisms) and those that enable plants to cope with Al^3^^+^ safely, once it enters the symplast (internal tolerance mechanisms; e.g., Kochian, 1995). Exclusion mechanisms depend on the release of ligands which chelate and detoxify Al^3^^+^ externally and limit its uptake in the cytosol. Tolerance mechanisms include those that chelate the Al^3^^+^ entering the root cells, with subsequent transport and sequestration into less sensitive parts of the plant and subcellular compartments. The physiology, biochemistry, and molecular biology of these mechanisms have been thoroughly discussed in several review articles (e.g., Jones and Ryan, 2003; Kochian et al., 2004; Ma, 2007; Poschenrieder et al., 2008; Horst et al., 2010; Ryan et al., 2011; Delhaize et al., 2012; Inostroza-Blancheteau et al., 2012). The proposed principles of these mechanisms are summarized in **Figure 2**.

In this paper, we review the literature on proposed Al^3^^+^ resistance mechanisms of woody plants. We summarize information obtained from crops and *Arabidopsis*, and then review relevant results from similar studies in woody plants. In addition, we discuss the occurrence of Al accumulators and Al excluders in different forest biomes of the world.

## Al^3^^+^ EXCLUSION

### RELEASE OF SUBSTANCES THAT CHELATE AND DETOXIFY Al^3+^

The best-documented mechanism of Al^3^^+^ exclusion is the Al^3^^+^-activated efflux of organic acids from roots. Typical organic acids released by plants are citrate, malate, and oxalate. These organic acids are deprotonated anions at the pH found in the cytosol, and once transported out of the root, they chelate the toxic Al^3^^+^ in the rhizosphere, forming stable and non-toxic complexes. Citrate and malate are present in all plant cells because they are involved in the mitochondrial respiratory cycle. Oxalate is a common cellular constituent involved in Ca^2^^+^ regulation, ion balance, and metal detoxification (Franceschi and Nakata, 2005; Rahman and Kawamura, 2011). The three organic acid anions form complexes with Al^3^^+^ with the following order of strength: citrate > oxalate > malate (Libert and Franceschi, 1987; Jones and Ryan, 2003). Physiological and genetic evidence from several plant species shows that the Al^3^^+^-activated efflux of organic acid anions indeed confers resistance to Al^3^^+^. The most convincing support comes from genotypes within a species that show contrasting levels of Al^3^^+^ resistance. In wheat (*Triticum aestivum*), for example, a pair of near-isogenic lines that differ in resistance at a single locus was used to show that the Al^3^^+^-activated efflux of malate from roots was greater in the resistant genotype than in the sensitive genotype (Delhaize et al., 1993). The same wheat genotypes were used to clone the Al^3^^+^-activated malate transporter (*TaALMT1*) gene, the first Al^3^^+^ resistance gene isolated from plants (Sasaki et al., 2004). This gene encodes a plasma membrane-bound protein responsible for the efflux of malate from roots. Subsequently, *TaALMT1*-like genes were isolated from several additional plant species, including *Arabidopsis* and rape (*Brassica napus*; Hoekenga et al., 2006; Ligaba et al., 2006; see also Delhaize et al., 2007). Citrate efflux, on the other hand, was found to be mediated by members of another protein family, the multidrug and toxic compound extrusion (MATE) family (Furukawa et al., 2007; Magalhaes et al., 2007). The molecular background of oxalate exudation has not yet been identified.

Numerous studies, some of which are described here, have found that woody plants release organic acid anions following Al^3^^+^ exposure. In the model tree poplar (*Populus*), the Al^3^^+^-activated release of organic acid anions has been studied both at the physiological and molecular level. In young rooted cuttings of *Populus tremula*, Al^3^^+^ induces the release of citrate and oxalate (Qin et al., 2007). In a follow-up study with the same poplar clone, Grisel et al. (2010) identified a *MATE* gene with a 60% amino acid sequence identity to the *AtMATE1* gene of *Arabidopsis.* The poplar gene is induced by Al^3^^+^ in both root and stem tissue, but not in the leaves, consistent with a function of this gene in the efflux of citrate from roots. In seedlings of two other poplar species, *Populus tremuloides* and *Populus trichocarpa*, Al^3^^+^ induced the exudation of citrate, malate, and oxalate from roots (Naik et al., 2009). In these species, organic acids accounted for 20–64% of the total C released upon Al^3^^+^ exposure (Naik et al., 2009). The minimal concentrations of Al^3^^+^ required to induce organic acid exudation in *Populus tremula* are between 50 and 100 μM Al^3^^+^ (**Table 1**; Qin et al., 2007). Using the same solution culture medium, similar threshold values were found in the two coniferous trees *Cryptomeria japonica* and *Pinus thunbergii* (Hirano et al., 2012; **Table 1**). Although these studies clearly demonstrate that Al^3^^+^ induces the release of organic acid anions from roots, direct physiological and genetic evidence for their role in Al^3^^+^ resistance has not been established. For example, it is not known whether the organic acid anions are released primarily from the root tip, which would be indicative for a role in Al^3^^+^ resistance.

**Table 1:** Organic acid release from roots of *Cryptomeria japonica* (μmol g^–1^ day^–1^_FW_), *Pinus thunbergii* (μmol g^–1^ day^–1^_FW_), and *Populus tremula* (μmol g^–1^_DW_) after exposition to Al^3^^+^ (according to Qin et al., 2007; Hirano et al., 2012).

**Tree species**	**Al^**3**+^ concen-tration (μM)**	**Citrate**	**Malate**	**Oxalate**
*Cryptomeria japonica*	0	0.08^a^	0.10^a^	0.14^a^
	100	0.39	0.14	0.49
	500	0.41	0.14	0.75
	1000	0.38	0.13	0.70
	*P* value^b^	ns	ns	*
*Pinus thunbergii*	0	0.03^a^	0.03^a^	0.47
	100	0.10	0.03^a^	1.08
	500	0.10	0.03^a^	2.93
	1000	0.11	0.03^a^	4.04
	*P* value	*	ns	**
*Populus tremula*	0	0.0^a^	0.0^a^	0.1
	50	0.8	0.0^a^	4.5
	100	1.9	0.0^a^	3.9
	200	18.4	0.0^a^	5.6
	500	20.5	0.0^a^	25.7
	1000	20.3	0.0^a^	18.8
	*P* value	***	ns	***

*^a^Below detection limit.*

^b^ANOVA: ***P < 0.001, **P < 0.01, *P < 0.05; ns, not significant.

Woody plant species vary considerably in the organic acid compounds they release in response to Al^3^^+^ exposure. Many species exude more than one organic acid anion, with various combinations of malate, oxalate, and succinate. In the broad-leaved deciduous and evergreen trees and shrubs assayed so far, citrate is the most common organic acid anion identified (**Table 2** and references therein). Of the five coniferous tree species analyzed, three released oxalate, and the two remaining citrate and succinate, respectively (**Table 2**).

**Table 2:** Al^3^^+^-activated release of organic acids from roots of woody plants.

**Plant species**	**Al^**3**+^-activated organic acids**	**Reference**
**Non-mycorrhizal roots; coniferous trees**
*Cryptomeria japonica*	Citrate, oxalate	Hirano et al. (2012)
*Picea abies*	Oxalate	Heim et al. (2001)
*Picea abies*	–	Eldhuset et al. (2007)
*Pinus sylvestris*	Oxalate	Ahonen-Jonnarth et al. (2000)
*Pinus thunbergii*	Citrate, oxalate	Hirano et al. (2012)
**Non-mycorrhizal roots; broad-leaved trees (deciduous)**
*Populus tremula*	Citrate, oxalate	Qin et al. (2007)
*Populus tremuloides*	Citrate, malate, oxalate, succinate	Naik et al. (2009)
*Populus trichocarpa*	Citrate, malate, oxalate, succinate	Naik et al. (2009)
**Non-mycorrhizal roots; broad-leaved trees and shrubs (evergreen)**
*Acacia auriculiformis*	Citrate, oxalate	Nguyen et al. (2003)
*Camellia sinensis*	Oxalate	Morita et al. (2011)
*Camellia sinensis*	–	Ishikawa et al. (2000)
*Cinnamomum camphora*	Citrate	Osawa et al. (2011)
*Citrus grandis*	Citrate, malate	Yang et al. (2011)
*Citrus junos*	Citrate	Deng et al. (2009)
*Citrus sinensis*	Citrate, malate	Yang et al. (2011)
*Eucalyptus camaldulensis*	Citrate, oxalate	Tahara et al. (2008)
*Eucalyptus camaldulensis*	Citrate, oxalate	Nguyen et al. (2003)
*Eucalyptus cloeziana*	Citrate	Silva et al. (2004)
*Eucalyptus dunnii*	Citrate, malate, oxalate	Silva et al. (2004)
*Eucalyptus globulus*	Citrate, malate	Silva et al. (2004)
*Eucalyptus grandis*	Citrate	Silva et al. (2004)
*Eucalyptus saligna*	Citrate	Silva et al. (2004)
*Eucalyptus urophylla*	Citrate, malate, oxalate	Silva et al. (2004)
*Melaleuca bracteata*	Citrate	Tahara et al. (2008)
*Melaleuca cajuputi*	Citrate, malate	Tahara et al. (2008)
*Melaleuca cajuputi*	Citrate, oxalate	Nguyen et al. (2003)
*Melaleuca leucadendra*	Citrate	Nguyen et al. (2003)
**Mycorrhizal roots**
*Picea abies*	Succinate	Heim et al. (2003)
*Picea abies*	–	Eldhuset et al. (2007)
*Pinus densiflora*	Citrate	Tahara et al. (2005)
*Pinus sylvestris*	Oxalate	Ahonen-Jonnarth et al. (2000)

*The references are divided into studies dealing either with non-mycorrhizal roots or with mycorrhizal roots.*

Little is known about other substances that may be released by roots to chelate Al^3^^+^. Proposed compounds include polypeptides, phenolic compounds, cyclic hydroxamates, and rhizodepositions in the form of mucilage (Jones and Ryan, 2003; Poschenrieder et al., 2008). In the tea plant, *Camellia sinensis*, Morita et al. (2011) observed beside of oxalate an increase of the release of caffeine, a phenolic compound, in response to Al^3^^+^ exposure. Phenolic compounds were also exuded in Al^3^^+^-treated *Eucalyptus camaldulensis* and two *Melaleuca* species (Nguyen et al., 2003).

### RAISING THE pH IN THE RHIZOSPHERE

According to Kochian et al. (2005), only one study to date has unequivocally demonstrated that raising the pH in the rhizosphere can protect plants from Al^3^^+^. For two distinct classes of Al^3^^+^-tolerant *Arabidopsis* mutants, it was shown that Al^3^^+^ resistance is mediated by the exclusion of Al^3^^+^ from the root either by exudation of malate and citrate (Larsen et al., 1998) or by H^+^ influx at the root apex (Degenhardt et al., 1998). The H^+^ influx resulted in an increase in the rhizosphere pH, and subsequently in a significant decrease in the Al^3^^+^ activity around the root tip. However, there is currently no evidence to support that this mechanism operates in *Arabidopsis* ecotypes. Whether the roots of woody plants make use of such a mechanism remains elusive.

### MODIFICATION OF Al^3+^ BINDING SITES IN THE CELL WALL OF ROOT CELLS

The cell wall of root cells has been suggested to be a site of both Al^3^^+^ toxicity and Al^3^^+^ exclusion (Horst et al., 2010). It has been determined that up to 90% of the Al^3^^+^ absorbed by roots can be localized to the apoplast (Kochian, 1995). The primary site of Al^3^^+^ binding is probably the pectin matrix, which is largely composed of homopolymers of galacturonic acid (Mohnen, 2008; Horst et al., 2010). Al^3^^+^ is known to bind far more strongly to pectin than Ca^2^^+^, whose binding to the cell wall is required for proper cell wall functioning (Franco et al., 2004). It has been proposed that Al^3^^+^ binds to the cell wall through a replacement of Ca^2^^+^, making the cell wall more rigid, and thus reducing its extensibility which is required for normal cell elongation (Tabuchi and Matsumoto, 2001).

Several studies have suggested that the pectin content and the degree of pectin methylation are important determinants of the amount of Al^3^^+^ that can bind to the cell wall of root cells. In maize (*Zea mays*), rice (*Oryza sativa*), and common bean (*Phaseolus vulgaris*), differences in the pectin content and/or the degree of pectin methylation were linked with Al^3^^+^ sensitivity/resistance (Eticha et al., 2005; Yang et al., 2008; Rangel et al., 2009). Al^3^^+^-resistant lines of all three plant species were found to have a higher degree of pectin methylation, and a lower cell wall Al content when compared to Al^3^^+^-sensitive lines, supporting a role for pectin methylation in Al^3^^+^ exclusion. A modulating role for the degree of pectin methylation is further supported by the finding that the expression of pectin methylesterase (PME), the enzyme responsible for the demethylation of pectin, was lower in Al^3^^+^-resistant lines than in Al^3^^+^-sensitive lines (Maron et al., 2008; Yang et al., 2008).

Current evidence, based on X-ray microanalyses, indicates that the apoplast is a major site of Al accumulation also in woody plants. In Al^3^^+^-treated seedlings of the conifer *Picea abies*, Al was found in both epidermal and cortical cells of the root tip (Heim et al., 1999). In both cell types, more than 88% of the total Al localized to the cell wall. In addition, it was observed that the amount of Ca in the cell wall of both cell types was much lower in Al^3^^+^-treated seedlings than in control plants, suggesting that Al^3^^+^ replaced Ca^2^^+^ at the exchange sites of the cell wall. These findings are further substantiated by results of a study conducted in *Picea abies* and *Populus tremula*, cultivated in a model ecosystem for 3 years (Brunner et al., 2008). In *Picea abies*, Al accumulated continuously over time in the cell wall of root epidermal cells, whereas in *Populus tremula*, Al accumulated in the cell wall of both root epidermal and cortical cells. In both species, Al did not accumulate intracellularly (**Table 3**).

**Table 3:** Al accumulation in fine roots of *Picea abies* and *Populus tremula* [Al concentrations of bulk material; Al net counts of compartments using , energy-dispersive X-ray spectroscopy (EDX)-analyses] after growth in weakly acidic soil (pH 6.5) with different length of exposition time (according to Brunner et al., 2008).

Tree species	Time (year)	Al concentration (mg g^–1^)	Al counts in epidermal cells		Al counts in cortical cells	
			Cell wall	Intracellular	Cell wall	Intracellular
*Picea abies*	0.5	2.12	213	83	126	94
	1.5	8.23	284	73	122	72
	2.5	9.50	355	101	140	80
	*P* value^a^	**	***	ns	ns	ns
*Populus tremula*	0.5	1.81	168	67	96	65
	1.5	16.40	359	50	152	51
	2.5	5.36	338	57	163	63
	*P* value	–^b^	***	ns	**	ns

^a^Repeated measures ANOVA: ***P < 0.001, **P < 0.01, *P < 0.05, ns, not significant.

*^b^No statistical analysis possible because of missing replicates.*

In root cells of woody plants, little evidence exists about the relationship between cell wall polysaccharides and Al^3^^+^ sensitivity/resistance. In a set of poplar clones, representing several interspecific crosses, it was found that the Al content of the root symplast was higher in Al^3^^+^-resistant clones than in Al^3^^+^-sensitive clones (Smith et al., 2011). The Al content of the root symplast, on the other hand, was lower in Al^3^^+^-resistant clones than in Al^3^^+^-sensitive clones. This pattern of cellular Al distribution suggests that the cell wall of root cells prevented Al^3^^+^ from entering the root symplast. Additional parameters investigated were pectin and callose, the latter of which is a widely used indicator of early Al^3^^+^ toxicity symptoms (Hirano et al., 2004, 2012; Kochian et al., 2005). Treatment with Al^3^^+^ increased pectin and callose levels in all clones, but more prominently in Al^3^^+^-sensitive clones. A clear conclusion about the impact of pectin could not be drawn because the degree of pectin methylation was not assessed.

## Al^3+^ TOLERANCE

### CHELATION OF Al^3+^ WITH ORGANIC SUBSTANCES IN THE CYTOSOL

Organic acid anions and phenolic compounds have also been implicated in internal Al^3^^+^ tolerance. Once Al^3^^+^ enters the cell, the concentration of free Al^3^^+^ cations in the cytosol will be very low, but even at these concentrations, Al^3^^+^ remains a hazard. The very high affinity of Al^3^^+^ for oxygen ligands allows it to compete with other ions for metabolically important sites despite a large disparity in their concentrations (Jones and Ryan, 2003).

Indeed, studies of several woody plant species demonstrate that intracellular Al^3^^+^ is chelated by organic acid anions. In the small shrub *Melastoma malabathricum*, upon entering the root, Al^3^^+^ binds to citrate, and the Al–citrate complex itself is transported from the root to the shoot (Watanabe and Osaki, 2001). In the leaves, the Al-citrate complex is transformed into Al–oxalate 1:1, 1:2, and 1:3 complexes. The former two complexes are potentially toxic to the plant. A similar transformation of Al–organic acid complexes is described for the tea plant. Upon entering the root cell, Al^3^^+^ binds to oxalate and then is transported from the root to the shoot in the form of Al–citrate and Al–malate complexes (Morita et al., 2004, 2008). In a comparison of several *Eucalyptus* species, the concentration of root tip malate was found to correlate positively with the degree of Al^3^^+^ resistance in the presence of Al^3^^+^ (Silva et al., 2004). In contrast, in the poplar clones of the above-mentioned study, the concentrations of symplastic citrate and formate correlated closely with Al^3^^+^ sensitivity (Smith et al., 2011).

Besides organic acids, there are other complex forming compounds, e.g., phenolic substances, that bind Al^3^^+^ in the cytosol. For example, in the tea plant, Al–catechin complexes were described (Nagata et al., 1992). In the sepals of the small shrub *Hydrangea macrophylla*, Al^3^^+^ is bound to both 3-caffeoylquinic acid and delphinidin 3-glucoside, where Al^3^^+^ is thought to play a role in stabilizing the two organic compounds, and thus causing the color to change from red to blue (Ma et al., 2001). In the root apices of the camphor tree (*Cinnamomum camphora*), an accumulation of proanthocyanidin, which is composed of flavan-3-ols (e.g., catechin), has been demonstrated (Osawa et al., 2011). An increase in root phenolics has been observed by Ofei-Manu et al. (2001) in a series of woody plants upon Al^3^^+^ exposure, including *Camellia sinensis*, *Cryptomeria japonica*, *E. viminalis*, *Gleditsia triacanthos*, *Picea abies*, *Pinus densiflora*, *Pinus thunbergii*, *Populus tremuloides*, *Robinia pseudoacacia*, and *Rhus succedanea.*

### SEQUESTRATION OF Al^3+^ TO METABOLICALLY LESS SENSITIVE COMPARTMENTS

The uptake and storage of high Al^3^^+^ concentrations in aerial parts of the plant is a trait common to many plant species of tropical regions, where the ability to cope with Al^3^^+^ stress is a strong prerequisite for survival (Ryan and Delhaize, 2010). Plants that accumulate >1 mg g^-^^1^_DW_ Al are considered Al-hyperaccumulators (Jansen et al., 2002). Plant families with woody Al-hyperaccumulators storing very large amounts of Al (>10 mg g^-^^1^_DW_) in their leaves include Melastomataceae, Rubiaceae, and Theaceae (Matsumoto et al., 1976; Watanabe et al., 1997; Masunaga et al., 1998; Jansen et al., 2003; Olivares et al., 2010; Gonzalez-Santana et al., 2012).

A typical example of a woody plant capable of accumulating large amounts of Al (>15 mg g^-^^1^) in its leaves is the tree species *Richeria grandis* from the Venezuelan cloud forest. Using X-ray microanalysis, Cuenca et al. (1991) showed that Al is stored extracellularly in the cell wall of mature leaves. Further extracellular cell wall storage of Al was demonstrated in the woody Al-hyperaccumulators *Camellia sinensis*, *Conostegia xalapensis*, *Faramea marginata*, and *Melastoma malabathricum.* The cell wall of the epidermal and the mesophyll cells of the leaves were the main sites for Al accumulation (Watanabe and Osaki, 2001; Britez et al., 2002; Tolra et al., 2011; Gonzalez-Santana et al., 2012). Interestingly, the chloroplasts of *Qualea grandiflora* and *Callisthene major*, two Al-hyperaccumulating woody plant species from the Vochysiaceae family, which grow in the Brazilian Cerrado, have been suggested as a primary compartment for Al sequestration (De Andrade et al., 2011). Al^3^^+^ can also be sequestered into cells specialized for storage functions, e.g., idioblasts containing Ca-oxalate crystals have been considered as a location for Al^3^^+^ detoxification in the leaves of *Corchorus olitorius* (Mazen, 2004).

**FIGURE 3:**
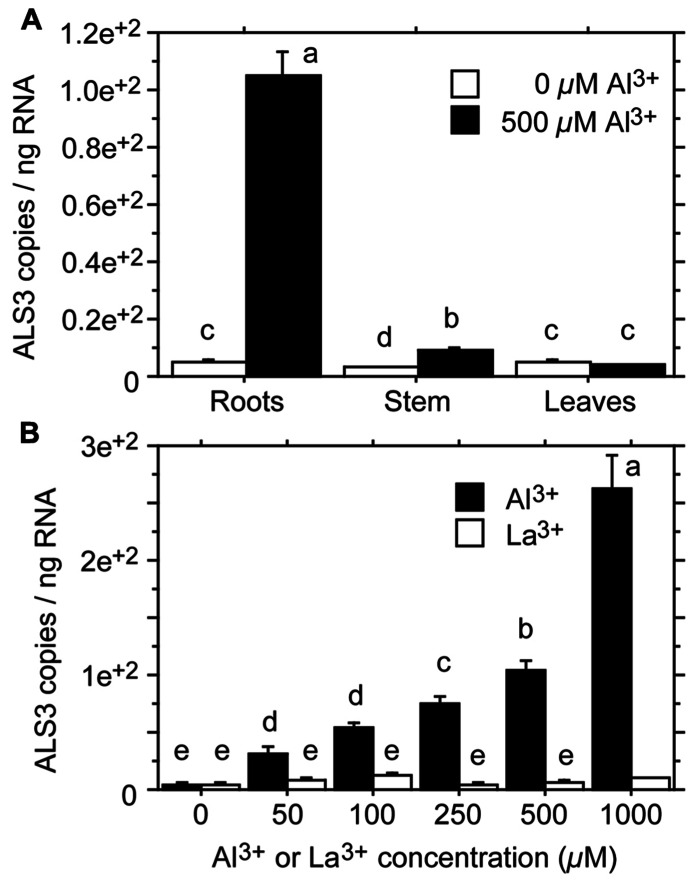
**Expression levels of *ALS3* in tissues of *Populus tremula* treated with Al^**3**+^ for 2 days.** **(A)** Expression levels in root, stem and leaf tissue after treatment with 0 or 500 μM Al^3^^+^. **(B)** Expression levels in the root tissue after treatment with 0, 50, 100, 250, 500, or 1000 μM Al^3^^+^ or La^3^^+^. Transcript levels were quantified by absolute qRT-PCR. Different letters indicate significant differences between treatments and elements: ANOVA; *P* < 0.05 (redrawn from Grisel et al., 2010).

Transport of Al from the root to the shoot is likely to involve complexes of Al with organic acids (see above). Little is known about the transport of Al across the plasma membrane and further sequestration into subcellular compartments. Transport across membranes requires transport proteins. Candidates for such proteins have been identified in the Al^3^^+^-sensitive mutants *als1* and *als3* of *Arabidopsis*. Both mutants are mutated in genes encoding proteins that belong to the ATP-binding cassette (ABC) transporter superfamily. ALS1 (Al^3^^+^-sensitive) is a half type ABC transporter, whereas ALS3 is an ABC transporter-like protein, lacking the ABC domain. The functions and substrates of ALS1 and ALS3 are not known, but the mutant phenotypes, the subcellular localization of the proteins, and tissue-specific gene expression have led to the assumption that the two proteins sequester and transport Al^3^^+^ to overcome Al^3^^+^ toxicity. ALS1 is likely to be involved in the intracellular transport of Al^3^^+^ to vacuoles of root tip cells and cells of the plant vasculature (Larsen et al., 2007). ALS3 is mainly localized to the plasma membrane of root cortex cells and phloem cells throughout the plant, suggesting that it mediates the intercellular redistribution of accumulated Al away from sensitive tissues (Larsen et al., 2005). In *Populus tremula*, Grisel et al. (2010) identified an *ALS3*-like gene with a 79% amino acid sequence identity with the *Arabidopsis* *ALS3* gene. The poplar gene was found to be expressed in the root, stem, and leaves, and was strongly induced by Al^3^^+^ in the root (44-fold; **Figure 3A**). In addition, the poplar gene was inducible by Al^3^^+^, but not by La^3^^+^ (lanthanum; **Figure 3B**), consistent with the finding that the *Arabidopsis* mutant *als3* is not affected by La^3^^+^ (Larsen et al., 1997).

### ACTIVATION OF METABOLIC PATHWAYS TO OVERCOME THE TOXIC EFFECTS OF Al^3+^

Moderate Al^3^^+^ concentrations are not fatal, and roots may at least partially recover (see also Matsumoto and Motoda, 2012). This is well documented in *Populus tremula* treated with either no Al^3^^+^ or increasing concentrations of Al^3^^+^ up to 1,000 μM. Two phases of root growth could be distinguished: a rapid Al^3^^+^-induced growth inhibition (within 6 h at Al^3^^+^ concentrations > 250 μM) and a subsequent phase of growth recovery (within 2 days at Al^3^^+^ concentrations í 500 μM; Grisel et al., 2010). The root growth of plants treated with 1,000 μM Al^3^^+^ further decreased. This pattern of root growth recovery may reflect the success of the roots in activating metabolic pathways to overcome the toxic effects of Al^3^^+^ and/or Al^3^^+^ resistance mechanisms. Matsumoto and Motoda (2013) suggested that the recovery of roots exposed to Al^3^^+^ is associated with the reduction of Al^3^^+^-induced oxidative stress. In *Populus tremula*, two genes of the oxidative stress pathway (a peroxidase gene and an alternative oxidase gene) were strongly induced upon Al^3^^+^ exposition after 6 h (>8-fold), while their expression decreased to control levels after 2 days (Grisel et al., 2010).

Two additional genes of *Populus tremula* that may play a role in root growth recovery encode CorA-like Mg^2^^+^ transporters (Grisel et al., 2010). These genes were induced up to fivefold by Al^3^^+^. The activity of a homologous CorA-like Mg^2^^+^ transporter from *Arabidopsis* was shown to be blocked by micromolar concentrations of Al^3^^+^, when expressed in bacteria (Li et al., 2001). In addition, the same CorA-like Mg^2^^+^ transporter alleviated Al^3^^+^ toxicity when overexpressed *in planta* (Deng et al., 2006). Mg^2^^+^ has been reported to be able to alleviate Al^3^^+^ toxicity in a number of crop plants (see reviews of Bose et al., 2011; Chen and Ma, 2013). Various mechanisms have been put forward to explain how Mg^2^^+^ can alleviate Al^3^^+^ toxicity. These mechanisms include increased ionic strength of the solutions, reduction in Al^3^^+^ saturation at the apoplastic exchange sites, and decreased Al^3^^+^ activity at the root cell plasma membrane surface (Bose et al., 2011). However, the identified Mg^2^^+^ transporters indicate that, besides of electrostatic interactions, biochemical processes may be involved in the rescue of Al^3^^+^ toxicity.

## ADAPTATIONS

The tropical forests and the forests of boreal and temperate regions have evolved on geological timescales under very different conditions. The forests of boreal and temperate regions were repeatedly affected by the Pleistocene glaciations, while the impact of the Pleistocene climatic fluctuations was certainly much less severe in tropical regions. Most tropical forests have not been disturbed for hundred thousands of years, and thus typically grow on highly weathered soils, which are strongly acidic, both in the topsoil and the subsoil (**Figures 1A,B**).

Accumulating evidence shows that in tropical regions both Al excluders and Al accumulators occur. Examples of woody species that are strong excluders are *Melaleuca cajuputi*, *Acacia mangium*, and *Leucaena leucocephala* (Osaki et al., 1997), which are all capable of exuding organic acid anions from their roots (see also **Table 2**). Examples of woody species that store high amounts of Al in leaves are *Melastoma malabathricum* and *H. macrophylla*. Other woody species, such as *Vaccinium macrocarpon*, store high amounts of Al in their roots (Osaki et al., 1997). Most Al-hyperaccumulator plants are shrub-type broad-leaved woody plants.

Phylogenetic analyses indicate that Al hyperaccumulation is a trait that has arisen a number of times, and this trait is scattered over more than 20 orders across about 45 families belonging to magnoliids (e.g., Laurales), eudicots (e.g., Proteales), rosids (e.g., Malpighiales, Myrtales), and asterids (e.g., Gentianales, Ericales; **Table 4**; Jansen et al., 2002). To date, Al accumulation in tall trees has only been found in a few tree species of the Euphorbiaceae (Osawa et al., 2013). The predominance of Al accumulators within non-flowering plants suggests that Al accumulation evolved early in the evolution of land-plants and is probably a primitive characteristic associated with survival in ancient Al^3^^+^-rich environments (Jansen et al., 2002).

**Table 4:** Families of woody plants with strong and/or numerous Al-hyperaccumulators (according to Jansen et al., 2002).

**Clade**	**Order**	**Family**
Magnoliids	Laurales	Lauraceae, Monimiaceae, Siparunaceae
Eudicots	Proteales	Proteaceae
Eurosids I	Malpighiales	Euphorbiaceae
Eurosids II	Myrtales	Crypteroniaceae, Melastomataceae, Vochysiaceae
Asterids	Ericales	Diapensiaceae, Symplocaceae, Ternstroemiacieae, Theaceae
Euasterids I	Gentianales	Rubiaceae

An analysis of the variation in foliar Al and macronutrient concentrations in a global dataset of plant species in a phylogenetic framework showed that the frequency distribution of foliar Al concentration in tropical regions clearly had two peaks (“bimodal”), whereas in temperate regions it showed only one peak (“unimodal”; Metali et al., 2012). This conclusion supports the hypothesis that Al accumulators and non-Al accumulators exist as distinct, but overlapping, groups of species. The estimated threshold value of foliar Al concentrations that distinguishes Al accumulators from non-Al accumulators varies geographically. The foliar threshold of tropical plants is higher (2.3–3.9 mg g^-^^1^) than that of temperate plants (1.1 mg g^-^^1^; Metali et al., 2012). Among angiosperm species, there was a significant phylogenetic signal in foliar Al concentrations, substantiating results of previous studies, suggesting a greater prevalence of Al accumulators in some families than in others (e.g., Jansen et al., 2002, 2003). A phylogenetical signal may also arise when related species occupy relatively similar habitats that differentially influence nutrient uptake and accumulation (Thompson et al., 1997). For example, Schreeg et al. (2010) have provided evidence for significant associations between high soil Al and Mn concentrations and the distributions of trees in the Vochysiaceae and Myrtaceae on a 50-ha plot in a semideciduous moist forest in Panama.

Compared to soils of tropical regions, soils of boreal and temperate regions are generally younger, although large areas, such as Siberia and Beringia, were ice-free during the Last Glacial Maximum (26,500–18,000 year before present; Clark et al., 2009). However, soils of these ice-free areas were severely affected by cold climate and permafrost, strongly limiting soil chemical and soil biological processes. As a consequence, soils in boreal and temperate regions are generally less weathered and thus less acidic than tropical soils, particularly in the subsoil (**Figure 1B**). In contrast, topsoils of the boreal regions are highly acid due to the strong acidifying effect of the coniferous litter during the incomplete decomposition process and the formation of humic acids (Schachtschabel et al., 1992).

Aluminum concentrations measured in roots from temperate or boreal woody species suggest, that the immobilization of Al^3^^+^ in the cell wall is most likely an important adaptation. Al concentrations in the fine roots of common temperate and boreal woody species usually exceed the limit for being hyperaccumulators (>1 mg g^-^^1^). For example fine roots of *Abies alba*, *Castanea sativa*, *Fagus sylvatica*, *Picea abies*, *Pinus cembra*, and *Pinus montana* all have values of 1–10 mg g^-^^1^_DW_ (Zysset et al., 1996; Brunner et al., 2002; Genenger et al., 2003; Hirano et al., 2006; Richter et al., 2011; see also **Table 3**). All these woody species are ectomycorrhizal, suggesting that ectomycorrhizal structures, such as fungal mantle and Hartig net, further contribute to the accumulation of Al in roots by immobilizing Al in the cell wall of the fungal hyphae (Brunner and Frey, 2000; Heim et al., 2003) or in the fungal vacuoles (Martin et al., 1994). Ectomycorrhizas in temperate and boreal regions have, we assume, the function not only to take up water and nutrients but also to immobilize toxic Al^3^^+^. Because ectomycorrhizal trees and ericoid-mycorrhizal Ericales dominate in temperate and boreal regions, we propose that this mechanism to immobilize Al^3^^+^ is a major mechanism for excluding Al^3^^+^ from the roots of woody plants in these regions, a suggestion first proposed by Jansen et al. (2002). Moreover, mycorrhizal fungi may well contribute significantly to the cycling of Al in forest ecosystem because high concentrations of Al can be found in their fruiting bodies (Smits and Hoffland, 2009). An additional adaptation of tree roots from temperate or boreal forests to acid soils is the observation that the roots have a shorter lifespan, which means that the turnover rate of roots exposed to elevated Al^3^^+^ concentrations is higher (Godbold et al., 2003; Leuschner et al., 2004; Richter et al., 2013). Reasons for a shorter lifespan could be that Al accumulation has reached its saturation point faster compared to roots from non-acid soils, causing the root to die earlier.

Some woody plants in temperate regions have not evolved strategies to deal with high levels of Al^3^^+^ and thus are not adapted to acid soils. Examples are the two species *Fraxinus excelsior* and *Acer pseudoplatanus*, which both are not ectomycorrhizal. They grow mainly on forest sites with high pH and high base saturation, and are unlikely to occur on very acid soils (Weber-Blaschke et al., 2002). In a recent study, Walthert et al. (2013) showed that *Fraxinus excelsior* and *Acer pseudoplatanus* respond much more sensitively to soil properties than *Fagus sylvatica* does. The soil properties limiting their distribution are Al in the case of *Fraxinus excelsior*, and Al together with nutrient availability (C/N ratio) in the case of *Acer pseudoplatanus*. On the contrary, no soil-induced distribution limits were detectable for *Fagus sylvatica* (Walthert et al., 2013).

Differences in Al^3^^+^ resistance not only exist among different tree species but also among local populations of the same species. Kidd and Proctor (2000) assessed the level of Al^3^^+^ resistance in four populations of *Betula pendula* growing on soils that differ in soil acidity and levels of Al^3^^+^. Using a solution culture system which simulates natural soil solutions, the authors found that seedlings originating from populations from acid mineral soils (pH 4.3, Al^3^^+^ concentration 21.1 mg l^-^^1^), had a significant higher level of Al^3^^+^ resistance (measured with the root elongation rate) compared to seedlings from populations from non-acid soils (pH > 4.8, Al^3^^+^ < 5.3 mg l^-^^1^). A similar study was performed by Wilkins and Hodson (1989) who analyzed two populations of *Picea abies* growing on an acid mineral soil and a calcareous soil, respectively. The growth of seedlings from the acid soil was slightly stimulated by the Al^3^^+^ treatment, whereas the growth of that from the calcareous soil was greatly reduced. The results of these studies suggest that high Al^3^^+^ soil conditions are a significant force for population divergence due to strong selective pressure associated with adaptation.

Further evidence for population adaptation to acid soil conditions comes from a study of *Pinus contorta* growing along a steep gradient of soil acidity at the northern coast of California (Eckert et al., 2012). In this area, five well-developed marine terraces exist, whose soils represent a chronosequence ranging from fertile soils close to the coast to podzolic soils with low pH and high Al^3^^+^ concentrations about 5 km inland (Westman, 1975). *Pinus contorta* is one of three conifers that have colonized and diversified on the extreme podzolic soils. Shore pine (*Pinus contorta* ssp. *contorta*) is found along the lower terraces, while Bolander pine (*Pinus contorta* ssp. *bolanderi*), which has a pigmy growth habit, is endemic to the upper marine terraces. Using a candidate gene approach, Eckert et al. (2012) investigated the molecular basis for the colonization of the podzolic soils by *Pinus contorta* populations. Patterns of nucleotide diversity were analyzed in genes that are related to growth or response to several soil parameters, such as Al^3^^+^, BC, and phosphate. The majority of the 21 genes analyzed did not or only weakly deviate from neutrality in patterns of nucleotide diversity. However, two of the genes carried clear signatures of positive selection: one was a putative homolog of the *Arabidopsis* *ALS3* gene and the other an inorganic phosphate transporter gene. The *ALS3* gene was characterized by a derived non-synonymous mutation, which is extremely rare in populations of the lower terraces and almost or completely fixed in those of the higher terraces. The phosphate transporter gene included two highly divergent haplotypes, whose frequency differed among lower and higher terraces. The results of this study shed light on some of the genetic components underlying adaptation to local soil conditions along this unique environmental gradient.

## CONCLUSION

Current evidence indicates that woody plants native to acid soils have evolved various strategies to overcome Al^3^^+^ stress. These strategies include exclusion of Al^3^^+^ from the root tip, probably through the release of organic acid anions. The formation of ectomycorrhizal structures, which are capable of accumulating Al in the cell wall of hyphal cells, may also be regarded as an exclusion strategy, reducing the degree of contact of root cells to Al^3^^+^. Internal strategies rely on the transport and sequestration of Al in aerial parts of the plant, where the Al may be stored in the cell wall of different leaf tissues, or in subcellular compartments, like chloroplasts or possibly vacuoles. Some woody plant species may also store high levels of Al in the cell wall of root cells. The biochemical and molecular mechanisms underlying these strategies, however, remain largely to be determined. The few studies performed so far in woody plants provide hints in which direction research may focus. With the exception of poplar, forest trees are generally not amenable to genetic engineering for testing the impact of candidate genes. Similarly, progenies of controlled crosses, which are valuable for testing segregation of specific traits and genes, exist only for a few tree species. An alternative approach would involve the identification of genes that play a role in adaptation through association of genetic variation with particular soil Al^3^^+^ conditions. This has already been initiated and has provided insights into the mechanisms of population adaptation to Al^3^^+^-rich environments.

## Conflict of Interest Statement

The authors declare that the research was conducted in the absence of any commercial or financial relationships that could be construed as a potential conflict of interest.
